# Video labelling robot-assisted radical prostatectomy and the role of artificial intelligence (AI): training a novice

**DOI:** 10.1007/s11701-022-01465-y

**Published:** 2022-10-30

**Authors:** Samy Cheikh Youssef, Nadine Hachach-Haram, Abdullatif Aydin, Taimur T. Shah, Nikhil Sapre, Rajesh Nair, Sonpreet Rai, Prokar Dasgupta

**Affiliations:** 1 MRC Centre for Transplantation, King’s College London, King’s Health Partners, London, UK; 2 Department of Plastic Surgery, Guy’s and St. Thomas’ NHS Foundation Trust, King’s Health Partners, London, UK; 3 Urology Centre, Guy’s and St. Thomas’ NHS Foundation Trust, King’s Health Partners, London, UK

**Keywords:** Computer vision, Robot-assisted radical prostatectomy, Artificial intelligence, Video labelling, Surgical video

## Abstract

Video labelling is the assigning of meaningful information to raw videos. With the evolution of artificial intelligence and its intended incorporation into the operating room, video datasets can be invaluable tools for education and the training of intelligent surgical workflow systems through computer vision. However, the process of manual labelling of video datasets can prove costly and time-consuming for already busy practising surgeons. Twenty-five robot-assisted radical prostatectomy (RARP) procedures were recorded on Proximie, an augmented reality platform, anonymised and access given to a novice, who was trained to develop the knowledge and skills needed to accurately segment a full-length RARP procedure on a video labelling platform. A labelled video was subsequently randomly selected for assessment of accuracy by four practising urologists. Of the 25 videos allocated, 17 were deemed suitable for labelling, and 8 were excluded on the basis of procedure length and video quality. The labelled video selected for assessment was graded for accuracy of temporal labelling, with an average score of 93.1%, and a range of 85.6–100%. The self-training of a novice in the accurate segmentation of a surgical video to the standard of a practising urologist is feasible and practical for the RARP procedure. The assigning of temporal labels on a video labelling platform was also studied and proved feasible throughout the study period.

## Introduction

Video labelling is the process through which different aspects of a video are assigned specific informative labels which a machine can utilise in machine learning and in the development of computer vision. Computer vision is the field of artificial intelligence (AI) which enables machines to develop a human-like understanding of different aspects of images and videos and enables the human-like learning of information from visual data. Machines to date can be taught to infer, analyse, and detect subtle data patterns and compute on their own through unsupervised machine learning, necessitating no explicit instruction [1].

One application of video labelling in surgery is the automatic segmentation of surgical videos. This application could be employed in the education of surgical trainees with research supporting the utilisation of video-based educational interventions [2, 3]. The automatic segmentation of surgical videos may also enable easy navigation through video indexing, improve interpretability of surgical video, and aid assessment of surgical skill [4, 5].

Although automation in surgical procedures is likely to remain out of reach in the near future, there is potential in the synergy between computer vision in AI and automation of surgical video labelling. A difficulty in the training of machine learning algorithms for classifying features of visual data is the prerequisite for large quantities of pre-labelled data [6]. Due to the nature of surgical videos and the heterogeneity in surgeon approaches, patient anatomy, and intraoperative events—to achieve accurate annotation, surgical expertise is necessary. However, the feasibility of incorporating experienced surgeons in such studies may not be feasible at every institution and is costly, both in their employment to perform such research and in time which could be spent treating patients [7].

Research for crowd annotation with surgical video is scarce, and current evidence suggesting a strong corelation between crowd annotators and clinical experts remains inconclusive for tasks of higher complexity or the labelling of full-length surgical procedures [7]. The established difficulties with labelling libraries of surgical video for computer vision research warrant further research into alternative methods in which accurately labelled video datasets can be produced. This study investigates the feasibility of training a novice student, whereby a foundational knowledge of anatomy and research is existent, in labelling a full-length robot-assisted radical prostatectomy (RARP) video.

## Methods

Over a period of 2 months, 25 RARP procedures performed on the Da Vinic Si HD dual console system were recorded on Proximie, a novel commercially available GDPR and HIPAA compliant augmented reality platform, approved by Guys and St. Thomas’ NHS trust (GSTT) IT department.

The footage was captured in the endoscopic view, producing a library of two-dimensional (2D) full-length RARP videos which were deidentified and anonymised. This video set was accessible via a secure, online, cloud-based storage which only the study participants had access to.

The student was trained on video labelling through self-directed learning, a review of the literature and reference to online video materials. Subsequently, a random video was selected from the dataset to be labelled on an online video labelling platform. This same video was then assessed according to an agreed pre-set checklist, on a 5-point Likert scale by four practising urologists experienced in performing RARP. The accuracy of video labelling was then calculated and documented.

### Patient and public involvement

Patients were not involved in the design and conduction of this study. Prostatectomy patients at GSTT undergoing RARP completed a standardised consent form for the storage and usage of surgical video. Surgical videos were than obtained and accessed via Proximie, a GDPR and HIPAA compliant platform.

### Data collection

The student was able to access the video through a secure online cloud-based storage with files protected by a 256-bit advanced encryption standard (AES). Patients had full knowledge of recording prior to the procedure and completed a consent form enabling the subsequent usage of the operative video. Videos were anonymised using an arbitrary numerical system deidentifying patients. A random number generator was utilised to select a video for assessment of video labelling accuracy [8].

### Video labelling

A comprehensive library of operative steps was outlined according to those previously defined in the literature, considering the steps which would be visible throughout operative video (robot setup and positioning, pneumoperitoneum, and port placement omitted as these steps are not visible in surgical video). The nine final steps assessed are outlined in Table 1. These steps were defined based on the review of relevant literature [9, 10], and were then validated for video labelling by a robotics clinical fellow and expert urologist.

**Fig. 1:**
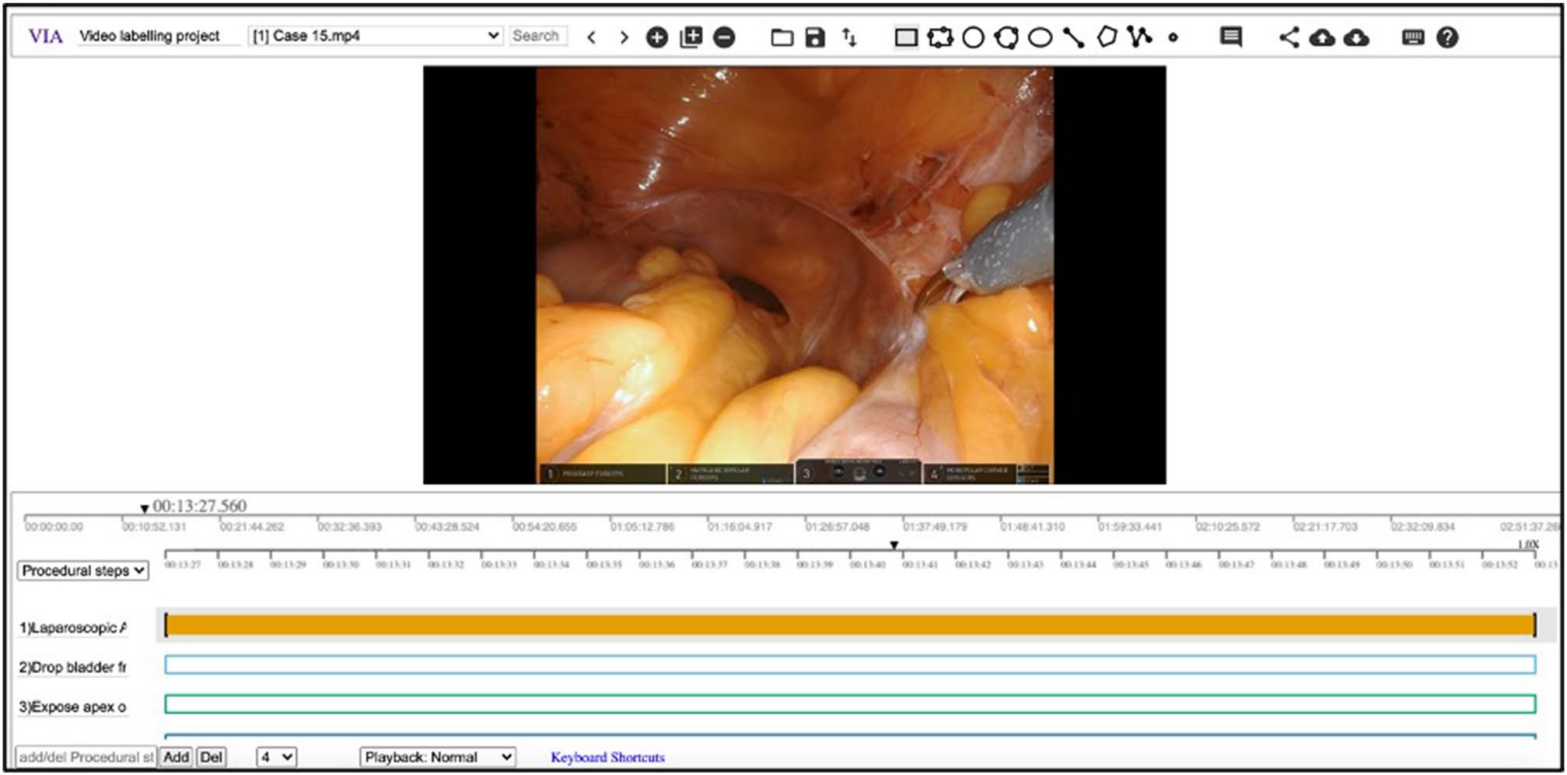
Video labelling platform used (VIA), 2D surgical footage, and procedural steps for labelling visible

Video labelling was performed on the VGG image annotator (VIA) platform [11] by the student (Fig. 1). For the purposes of review, operative video was disseminated via a cloud-based storage network using a private link, and time stamps were listed and sent separately as a word document.

### Assessment of accuracy

Following the learning period, a video amongst the data set was randomly selected [8] to test the video labelling accuracy of the student. The accuracy was graded on a five-point Likert scale [12] and completed forms were collected from the participants, the mean scores were calculated for the 9 procedural steps (Tables 1 and 2).

#### Procedural steps for video labelling

See Table 1.

**Table 1:** RARP steps used for identification in surgical video

Procedural step	Start and end
(1) Laparoscopic adhesiolysis	• **Start:** Insertion of working ports, lysis of abdominal adhesions + lysis of pelvic adhesions until the sigmoid colon using 4th arm for retraction • **End:** When peritoneal incisions begin
(2) Drop bladder from anterior wall	• **Start:** Peritoneal incisions • **End:** When pre-vesical space of Retzius is visualised and median umbilical ligament incised
(3) Expose apex of prostate	• **Start**: Removal of fat from puboprostatic ligaments, incision of endopelvic fascia • **End:** incision of puboprostatic ligaments
(4) Stitching of DVC (Varies—usually later)	• **Start:** Suture DVC • **End:** Dissect DVC
(5) Bladder neck transection	• **Start:** Bladder transection by 4th arm/stay suture, incision of anterior bladder neck at prostate vesical junction, Foley catheter usually visualised here • **End:** Posterior bladder neck transected
(6) Seminal vesicle dissection and retraction	• **Start:** Identification of seminal vesicles and vasa, dissection of vasa and seminal vesicles and retraction of seminal vesicles anteriorly and cranially, Visualisation of Denonvilliers fascia and development of rectal plane • **End:** prostate Is mobilised from rectum
(7) Dissection of prostate and neurovascular bundle	• **Start:** Sparing of neurovascular bundle from prostatic capsule (Varies) through incision of lateral prostatic fascia, clipping of neurovascular pedicles, • **End**: dissection of neurovascular bundle and freeing of prostate
(8) Apical dissection of prostate	• **Start:** incision of puboprostatic ligaments and suturing of the DVC (if not already done) • **End:** division of urethra
(9) Vesicourethral anastomosis	**Standalone**

## Results

### Quality of surgical video

**Fig. 2:**
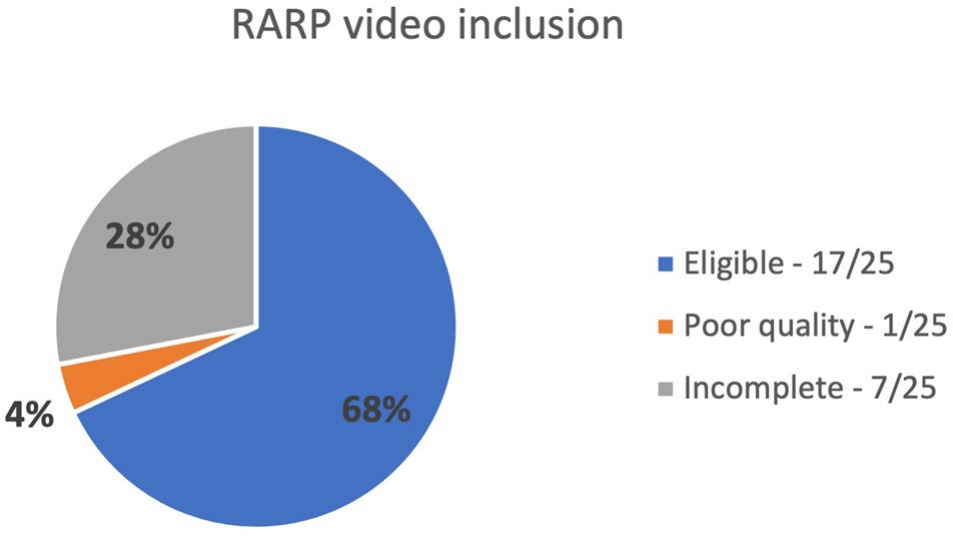
Classification of video dataset quality to determine eligibility for video labelling

Of the 25 analysed videos, 8/25 (32%) videos were deemed incomplete/low quality, and these videos were not used for review or labelling by the student due to the operative video being less than 1 h in length (incomplete), missing significant steps or the video being pixelated and unclear to the viewer. Of the 17 videos which were deemed to be of sufficient quality for analysis, videos were time stamped by the student as part of the learning process (Fig. 2).

### Accuracy of video labelling

The annotated video then underwent review by the four participating urologists, who then quantified the accuracy of labelling of each procedural step, over a 5-point Likert scale. The mean accuracy of the labelled video across all steps was 93.06%, with a range from 85.6 to 100%. Refer to Table 2 for complete data.

#### Accuracy scores

See Table 2.

**Table 2:** Accuracy scores—accuracy scores less than 60% (on average less than neutral overall) are deemed inaccurate on the merit of the proposed scoring system

Reviewer + stage of training		Operative step									Overall accuracy
		1	2	3	4	5	6	7	8	9	
NS (post-CCT clinical fellow)	Score of operative steps (out of 5)	5.00	5.00	5.00	5.00	5.00	5.00	3.00	5.00	5.00	43.00/45.00 95.60%
SR (post-CCT clinical fellow)		5.00	5.00	5.00	5.00	5.00	5.00	5.00	5.00	5.00	45.00/45.00 100.00%
TS (post-CCT clinical fellow)		5.00	3.00	4.00	5.00	2.50	4.00	5.00	5.00	3.00	38.50/45 85.60%
RN (consultant urological surgeon)		5.00	5.00	5.00	4.00	4.00	5.00	4.00	4.00	5.00	41.00/45.00 91.11%
Average scores		5.00	4.50	4.75	4.75	4.13	4.75	4.25	4.75	4.50	41.88/45.00 Mean = 93.06%

## Discussion

This purpose of this pilot study is to investigate the feasibility of video labelling by a novice student, as an initial step towards the development of an AI algorithm, automating the process of video labelling in RARP as previously done for laparoscopic sleeve gastrectomy [13], laparoscopic sigmoidectomy [4], and laparoscopic cholecystectomy [5].

The results of this study demonstrated the feasibility of a novice student to train in the labelling and accurate segmentation of operative video over a short-term period on a part-time basis; in this case, the robotic prostatectomy procedure performed primarily via a transperitoneal approach. A procedural video amongst the data set was then selected for review by four practising urologists. The results demonstrated an average of over 90% accuracy in the time stamping and video labelling of the procedural steps. The step which had the lowest accuracy score was step 5 (Bladder neck transection, see Table 2) at 4.13/5.00, which post-assessment feedback suggested was resultant to a misunderstanding of the relevant surgical anatomy. This finding suggests the potential utility in a standardised pre-labelling training programme, delivered by the practising surgeons, to the novices intending to perform video labelling. During such training, complex anatomy, potential anatomical variants, and operative steps which may incite confusion can be identified and novices can develop a more comprehensive understanding prior to the commencement of video labelling.

For the purposes of this study, the VIA annotation software [14] was not used by the assessors in grading the accuracy of the labelling; rather, the assessors were sent the surgical video via a collaborative cloud link which allowed access to the full-length case, accompanied by typed time stamps. The justification for this was the inconvenience which would be incurred if VIA were to be accessed to review a 3-h-long procedure. All the participants intending to review the surgical video with the temporal labels would be required to download the video which occupied 4.32 gigabytes (GB) and the alternative of a shared link was deemed sufficient in the context of this study and for review of the accuracy of time stamping.

The fields of surgical education, robotic surgery, and innovation in surgical technology with artificial intelligence are constantly evolving in the light of new technological developments. The video labelling performed in this project has several applications. In the immediate context, the organisation of a video dataset into a cloud storage network and its segmentation into the constituting steps of the procedure can be used for: research purposes, increasing efficiency of post-operative review of surgeon performance, and educational purposes whereby clarity of video materials is possibly improved through the addition of time stamps [15]. The educational value of video-based education in surgery has been established in recent years [3, 16]. As for the educational impact of adding labels to surgical video, this will require the conduction of future randomised-controlled trials. With the reduced working hours, the increasing financial strains faced by the NHS and reduced exposure to core surgical procedures by core surgical trainees—a high-quality surgical video library across specialties may augment current surgical training and practise [17, 18]. The COVID-19 pandemic has also caused significant disruption to surgical training, with reports of substantially reduced operative experience [19, 20], and the employment of alternative teaching methods could prove advantageous given the ability to remotely learn from video-based resources.

Labelled video has multiple further applications, for example, the development of context aware operating rooms with surgical workflow analysis [4, 21–23], labelled video is required as an initial reference point for machine learning and the establishment of algorithms which automate labelling and segmentation of surgical video. The future prospect of video labelling algorithms lies beyond the simple segmentation of surgical video, and is directed towards higher reasoning functions, such as surgical skill feedback, analysis of operative skill metrics, and intraoperative clinical decision support [13, 15].

However, an inherent difficulty encountered with this process is the time and expertise investment due as part of the manual labelling process, often demanding the participation of expert surgeons or motivated trainees [13, 15, 24]. This is where potential lies for student participation, and the current study has proven the possibility of student participation in a video labelling project. With numerous studies suggesting high interest to participate in research projects amongst students [25, 26], it may be an affordable, feasible alternative of benefit to both students and researchers.

Though this study has limitations. The results of this project may not be generalisable to all other endoscopic procedures. The results suggest promise in the ability of a student to learn to accurately identify the procedural steps within a surgical video, and subsequently label these. However, replicating this study for other procedures may demonstrate a difference in learning curves. Another significant feature of this study is that the data set was obtained from a single hospital, and the approach taken in performing RARP procedures in other hospitals may not be representative. It is known that there are several dissection techniques which may be done for RARP, with each differing in the anatomical structures encountered, the ability for labelling through different anatomical approaches was not tested in this study [27].

The context in which this study was conducted was during the peak of the COVID-19 pandemic, where clinical responsibilities of the participant surgeons and contact restrictions limited the recruitment of a student cohort. Despite the limitations, this study serves as a pilot study for more comprehensive future video labelling research. A formalised educational process can be applied amongst a cohort of students, to determine interrater reliability for students who have undergone the same educational process.

### Implications for future research

Advancements in AI and surgical technology have prompted further research studies. A barrier to the conduction of such studies can often be due to the requirement of high expertise and associated funding. This study has shown that a B.Sc. student, over the course of 2 months, was able to self-train in the understanding and accurate segmentation of the RARP procedure with no prior exposure to robotic surgery, having analysed and manually labelled an entire dataset. This could form the basis for an educational surgical video library, a reference point for assessors, however most applicable to this project, the use of video datasets in computer vision research and AI applications. Studies assessing the ability of students to perform such roles are scarce in the literature and may be of benefit to the scientific community due to the existent interest for research participation in students.

## Data Availability

No additional data available.
